# Nature and human well-being: The olfactory pathway

**DOI:** 10.1126/sciadv.adn3028

**Published:** 2024-05-15

**Authors:** Gregory N. Bratman, Cecilia Bembibre, Gretchen C. Daily, Richard L. Doty, Thomas Hummel, Lucia F. Jacobs, Peter H. Kahn, Connor Lashus, Asifa Majid, John D. Miller, Anna Oleszkiewicz, Hector Olvera-Alvarez, Valentina Parma, Anne M. Riederer, Nancy Long Sieber, Jonathan Williams, Jieling Xiao, Chia-Pin Yu, John D. Spengler

**Affiliations:** ^1^ School of Environmental and Forest Sciences, University of Washington, Seattle, WA 98195, USA.; ^2^ Department of Psychology, University of Washington, Seattle, WA 98195, USA.; ^3^ Department of Environmental and Occupational Health Sciences, University of Washington, Seattle, WA 98195, USA.; ^4^ Institute for Sustainable Heritage, University College London, London, UK.; ^5^ Natural Capital Project, Stanford University, Stanford, CA 94305, USA.; ^6^ Department of Biology, Stanford University, Stanford, CA 94305, USA.; ^7^ Woods Institute, Stanford University, Stanford, CA 94305, USA.; ^8^ Smell and Taste Center, Department of Otorhinolaryngology: Head and Neck Surgery, University of Pennsylvania Perelman School of Medicine, Hospital of the University of Pennsylvania, Philadelphia, PA 19104, USA.; ^9^ Interdisciplinary Center Smell and Taste, Department of Otorhinolaryngology, Faculty of Medicine Carl Gustav Carus, Technische Universität Dresden, Dresden, Germany.; ^10^ Department of Psychology, University of California, Berkeley, Berkeley, CA 94720, USA.; ^11^ Department of Experimental Psychology, University of Oxford, Oxford, UK.; ^12^ Wildwood/Mahonia, Portland, OR 97239, USA.; ^13^ Institute of Psychology, University of Wroclaw, Wrocław, Poland.; ^14^ School of Nursing, Oregon Health and Science University, Portland, OR 97239, USA.; ^15^ Monell Chemical Senses Center, Philadelphia, PA 19104, USA.; ^16^ T.H. Chan School of Public Health, Harvard University, Boston, MA 02115, USA.; ^17^ Air Chemistry Department, Max Planck Institute for Chemistry, 55128 Mainz, Germany.; ^18^ Climate and Atmosphere Research Center, The Cyprus Institute, Nicosia, Cyprus.; ^19^ College of Architecture, Birmingham City University, Birmingham, UK.; ^20^ School of Forestry and Resource Conservation, National Taiwan University, Taiwan.; ^21^ The Experimental Forest, College of Bio-Resources and Agriculture, National Taiwan University, Taiwan.

## Abstract

The world is undergoing massive atmospheric and ecological change, driving unprecedented challenges to human well-being. Olfaction is a key sensory system through which these impacts occur. The sense of smell influences quality of and satisfaction with life, emotion, emotion regulation, cognitive function, social interactions, dietary choices, stress, and depressive symptoms. Exposures via the olfactory pathway can also lead to (anti-)inflammatory outcomes. Increased understanding is needed regarding the ways in which odorants generated by nature (i.e., natural olfactory environments) affect human well-being. With perspectives from a range of health, social, and natural sciences, we provide an overview of this unique sensory system, four consensus statements regarding olfaction and the environment, and a conceptual framework that integrates the olfactory pathway into an understanding of the effects of natural environments on human well-being. We then discuss how this framework can contribute to better accounting of the impacts of policy and land-use decision-making on natural olfactory environments and, in turn, on planetary health.

## Teaser

A conceptual framework that integrates the olfactory pathway into research on nature and human well-being is presented.

## INTRODUCTION

The olfactory system has evolved to detect a vast range of airborne chemicals (*1*, *2*). These molecules, known as odorants, act as a type of volatile intermediary. They are emitted from a source, transported through the air, and provide information to an organism via olfactory perception (*3*). Odorants vary in their physiochemical properties, including molecular structure, functional groups, vapor pressure, and solubility (*4*, *5*) (see Box 1). In addition to differences in individual molecules, the trace gas composition of air that human beings breathe contains perpetually changing ratios and concentrations of these chemicals. This results in a range of olfactory environments that, while invisible, are dynamic, potentially highly potent, and consistently experienced by human beings to varying degrees of awareness (*6*).

Most living organisms rely on chemical senses (including the olfactory, gustatory, and trigeminal systems) for critical information about their environment. These influences span from spatial navigation to dietary choice to social organization (*7*). In mammals, after odorants bind to ciliated surfaces of olfactory receptor neurons located in the upper recesses of the nasal cavity, action potentials are generated that propagate along these neurons to the olfactory bulbs. From there, information is sent to brain structures critical for memory, affect, and a range of behaviors, including emotional responses mediated by the amygdala and other parts of the limbic system (*8*, *9*). A complex interacting neural network is involved in these processes, including important associations with the hippocampus and the orbitofrontal and dorsolateral prefrontal cortex (*10*–*13*).

Box 1 **Anatomical substrates, molecular underpinnings, and evolutionary function.**

**Molecular structure**

A current debate in the olfaction literature is the degree to which variance in perception is explained by the structure and composition of the odor molecule itself (*4*, *99*, *230*, *231*). Much is still unknown regarding the ways in which the brain creates and/or draws meaning from smells, and how these meanings vary (or not) according to structural diversity, composition, and other molecular features (*6*, *232*). Methods and insights from organic chemistry provide a substantial amount of knowledge regarding the intrinsic properties (e.g., chemoinformatic features) of volatile compounds and their reaction products, but an understanding of the underlying biological and psychological processes related to human perception of these molecules is still evolving (*8*, *44*, *233*–*237*).

Odorous compounds can be measured and described consistently, but the character and dimensions of quality, intensity, and hedonic tone that follow from their processing by the olfactory system depends at least in part on the specific nose, brain, and experiences of the individual perceiving them (*5*, *75*, *238*–*242*). Nonetheless, substantial progress has been made in understanding the basic elements of olfactory transduction, including the identification of gene families that express olfactory receptors (*243*).

**Evolutionary function**

Olfaction provides a major input to the limbic system, which subserves multiple brain functions in mammals, primarily memory and emotion (*9*, *58*). This system is responsible for the primary affective responses that vertebrates have to their environments, and this critical survival function is reflected in its evolutionary history and development (*58*, *244*–*246*). Along with influences on affect, olfaction plays a pivotal role in spatial orientation to odor plumes and trails (*247*) and has influenced hippocampal evolution (*248*–*253*). In these and other ways, olfactory cognition is deeply embedded in extended, complex olfactory landscapes (*244*).

## OLFACTION AND HUMAN WELL-BEING

Human beings typically have an excellent sense of smell, even when compared to a number of other animals, including mice and some canids (*14*–*17*). Yet, olfaction has been undervalued as a sensory pathway for human experience in Western cultures (*14*, *18*, *19*). Recent studies found that a sample of adults in the UK valued their sense of smell less than vision, hearing, touch, and taste (*20*), and many US college students from a separate sample stated that they would rather lose their sense of smell than their phone or hair (*21*). In addition, linguistic analyses of the most common word choices for descriptions of perceptual experiences reveal that they are often dominated by those related to vision (*22*, *23*).

Independent of judgments regarding its utility, however, it is clear that human beings relate to surrounding environments in important ways through olfaction (*10*). This is particularly salient with a global perspective. For example, odors found in nature play an important role in many Indigenous cultures around the world (*24*, *25*). A nomadic hunter-gatherer group from southern Thailand known as the Maniq routinely use smell to make foraging decisions and judge the medicinal properties of herbs. For the Maniq, smells are directly related to their sources, so unpleasant odors can be indicative of danger while pleasant ones are perceived to be protective and beneficial (*26*). Among a range of diverse communities across the globe and throughout different periods of history, odor has been considered a marker of health as well as individual and group identity and status—and is also closely tied to other aspects of social interactions, including friendship and marriage (*27*).

In addition to providing crucial contextual information, the human well-being impacts associated with olfactory processing of odorants are substantial (*28*). Olfactory function is associated with quality of and satisfaction with life (*29*–*33*). The sense of smell is tightly related to the limbic system (which evolved from the olfactory cortex) and to psychological processes (e.g., associations and memories) that play an integral role in everyday human functioning (*34*–*36*). This sensory system influences emotions, emotion regulation, cognitive function, social interactions, dietary choices, stress responses, and depressive symptoms, along with other core dimensions of life (*10*, *28*, *37*–*40*).

Volatile organic compounds (VOCs) and other airborne molecules can affect well-being through two primary olfactory pathways: (i) conscious (i.e., suprathreshold) perception of odors at varying degrees of awareness caused by the nervous system processing of these molecules (thereby classifying them as “odorants”) and (ii) nonconscious (i.e., subthreshold) processing of these molecules by the olfactory system that an individual does not perceive. These pathways may interact in complex ways—pleasant associations with smells that are physiologically harmful may provide a positive affective experience (*41*). For example, a positive childhood memory of woodsmoke associated with social connection can simultaneously produce adverse inflammatory effects via neuroimmunological or respiratory reactions (*42*). Similar complexities can exist with the use of scented candles (*43*). Distinguishing affective and physiological responses to airborne molecules via the olfactory pathway is therefore critical, as well as assessing aspects of the perceptions of odorants that take place at various levels of awareness (see Box 2).

Box 2 **Odor perception.**

For detection of odorants to be possible, the concentration of volatile molecules must exist at or above a threshold level for an organism. In human beings, biological, cultural, demographic, and environmental (both physical and social) factors affect the ability to detect and identify odors over time (*18*, *254*–*259*). Certain odorants bind to nociceptors on the trigeminal nerve and typically give rise to sensations of pungency (e.g., spices) or irritation (e.g., vinegar or acrolein). These sensations, combined with signals carried on the olfactory nerve, contribute to the perception of odor as well. “Olfactory sensitivity” refers to an organism’s ability to detect very low odorant concentrations. Operationally, it is defined as the lowest concentration of an odorant that an individual can consciously perceive (*260*). Age, gender, bodily state, learned behaviors, and abilities regarding directed attention to smell influence olfactory thresholds and, thus, awareness of an odor. Relevant environmental factors include ambient air pollution, concentrations and rates of change of airborne chemicals, the degree to which they are distinguishable from the background, humidity, distance, and other elements of a given geographic location (e.g., altitude) (*116*). ”Olfactory discrimination” is the process of distinguishing smells from each other without the requirement of identifying the odor. Olfactory discrimination can occur between chemicals at concentrations too low to be readily identified, i.e., where only a nuance is perceived, as well as between chemicals at higher concentrations where distinct odor experiences arise (*135*, *259*–*263*). ”Olfactory identification” describes the ability to associate an odor with a name and is largely dependent on language and experience (*24*, *264*). ”Odor memory” is the ability to recall experiencing an odor and is critical for odor perception, since, without memory, one cannot identify or discriminate between odors. Measures of odor detection, discrimination, identification, and memory are not mutually exclusive and often depend on the same underlying physiological substrates (*265*). For example, if sensitivity is markedly decreased, then the ability to identify and remember odors is also compromised. On the other hand, in some disorders such as chronic rhinosinusitis, odor sensitivity can be decreased while odor identification is still intact (*174*).

**Awareness**

Conscious awareness of odors is not a sufficient and necessary condition for many adaptive, olfactory-mediated functions (approaching, avoiding, and navigating) (*10*, *64*). Subconscious olfactory processing of odorants may also influence physiology, mood, behavior, cognition, and social interactions (*16*, *151*, *235*, *266*, *267*). One primary function of olfaction may be to notice changes in the environment and to take appropriate action in response to this perception (*268*, *269*). These phenomena can take place in an ongoing, continuous manner, even if explicit appraisal is missing until change occurs (*16*). In this way, human beings can be aware of smells without paying attention to them—an arena of experience that lies in between subconscious processing and explicit appraisal but that nevertheless has repercussions for a “state of being” (*10*). As another example, the olfactory vector hypothesis posits that a category of subthreshold impacts includes effects from aerosolized toxins, xenobiotics, and viruses that can “enter the brain via the nose”—traveling directly from peripheral tissue to the CNS and leading to neuroinflammation and other adverse effects (*270*). Olfactory receptor neurons are unique in their high level of exposure to the environment, providing a pathway through which volatile molecules can cause neuroinflammation through bypassing the blood-brain barrier (*271*, *272*).

In general, there appears to be a core universal bedrock to olfactory processing—an initial affective component that is prelinguistic, preceding cognitive appraisal but still based on suprathreshold perception (*18*, *19*, *44*, *45*). This early, preverbal affective reaction is likely independent of later processing that is informed by top-down processes and subjective experience. The latter category of appraisals contributes to evaluation and judgment about odor (un)pleasantness and is influenced by preference, culture, associations, prior experience, multisensory context, and other factors. These secondary responses to odors can also vary according to culture and individual-level factors such as genetics, age, and gender (*18*, *46*). In this way, the “hard-wired” spectrum of (un)pleasantness that exists for initial affective reactions to odors may differ from higher-order responses that are open to influence from cognitive processes (e.g., preference for the smell of rotten meat is modulated by culture) (*47*).

## NATURE, OLFACTION, AND HUMAN WELL-BEING

Whether in ancient or modern times, in rural hinterlands or megacities, human beings have always lived in complex interrelationships with nature. The importance of this relationship is emphasized in Indigenous Knowledge and by research in environmental anthropology (*48*–*53*). Along with these perspectives, Western-based knowledge systems and approaches to conservation have noted the degree to which we depend on tangible and intangible benefits from nature, some coproduced by people, to survive and flourish (*54*–*56*). However, much of the Western research specific to the impacts of nature contact on human well-being has focused on the causal mechanisms tied to the visual system (*57*, *58*)—including the foundations of influential theories in environmental psychology (*59*, *60*). These theories have motivated hypotheses and study designs in nature and health (*61*), such as a highly influential study on the impacts of views from a hospital window on recovery after surgery, for example (*62*). This perspective extends to much of psychology research in general, which often focuses on the visual versus other sensory pathways as well (*63*, *64*).

Here, we expand these considerations to include the olfactory pathway. This is a unique and important factor in the relationship of nature with human well-being, especially given the evolutionary history of olfactory responses to natural environments. There are human subjective olfactory experiences and affective responses that are specific to the natural world (*58*). These come from potted plants and private gardens, public greenspaces, the sea, wilderness, and other natural areas. Sailors crossing vast ocean expanses report smelling land long before seeing it. Smells of nature may be tied to an individual’s sense of place and call to mind associations or memories of specific natural landscapes (*65*).

These phenomena have been studied through a variety of methodologies. For example, in-person interviews centered on the sensory experiences of participants during walks have been used to assess smellscapes (i.e., the aspects of the environment that are perceived by human beings as smells, influenced by past experiences and spatial context, and that may affect individuals’ thoughts and feelings) (*66*–*68*). This approach has often focused on the built environment (*67*–*72*) and is now being applied to gardens, woodlands, and other natural environments (*73*, *74*).

Research in olfactory heritage emphasizes aspects of smells that are related to cultural practices and spaces with unique and integral values for identity and place-making for communities (*46*, *69*, *75*). This includes the smells of nature (*76*, *77*). For example, Sakura Blossom sites across Japan and lavender fields in France produce distinctive smells with acknowledged local significance and aesthetic values. These connections are related to the concept of olfactory heritage insofar as they are tied to dimensions of place-based identity for groups and individuals (*46*).

Other studies, predominantly from East Asia, demonstrate that the “immune system” of trees (i.e., the activation of volatile-mediated plant defenses to repel herbivores and pathogenic microbes) (*78*, *79*) may have an impact on the immune system of human beings. Shinrin-yoku (also known as “forest bathing”) researchers have investigated whether exposures to VOCs produced in natural environments confer psychophysiological and neuroimmunological benefits through the olfactory pathway (*80*–*82*). These studies focus primarily on chemical compounds known as terpenes that are naturally present in forests and have been found to be associated with short-term health outcomes in human beings (*82*, *83*), including impacts on mood, stress, anxiety, and inflammation (*80*, *84*, *85*). It is not yet known whether these effects are due to conscious appraisals of smells (e.g., the aroma of pine) and/or to neuroimmunological responses that bypass awareness (e.g., anti-inflammatory biochemical processes specific to terpene exposures).

This emerging body of research on the ways in which natural environments affect human well-being through the olfactory pathway informs the consensus statements below. Bringing together expertise across the health, natural, and social sciences, we offer the statements and the conceptual framework that follows as a foundation upon which future research can expand—as a more complete understanding is built regarding the relationship between nature, olfaction, and human well-being. When considering natural olfactory environments, we include VOCs and other airborne molecules produced by nature that are classified as odorants (i.e., perceived as odors), as well as VOCs and other airborne molecules from nature that bypass conscious awareness but still influence human well-being through the olfactory system.

## CONSENSUS STATEMENTS

### #1: Human beings are embedded in complex, rich, and prolific olfactory environments—chemical contexts within which the natural world transmits information

Volatile chemical emissions convey a multitude of signals both within and between plants and animals—a key function for life in the natural world (*86*, *87*). Olfactory environments play an essential role in microbial, plant, and animal communications (*88*–*90*). Plants emit odorous volatiles that are crucial for defense against herbivores, pollination (reproduction), and animal navigation (*79*, (*87*, (*91*–(*94*). These compounds are then transformed through oxidation, increasing molecular diversity as the concentrations of primary emissions decay (*95*). Human beings are embedded in these contexts, likely affect them through the generation of our own oxidation fields (*96*), and yet consciously perceive only a small proportion of the total chemosensory communication that exists within them (*5*).

### #2: Airborne chemicals from the natural environment affect human well-being through pathways specific to olfaction—initially perceived and later explicitly judged through both innate and acquired processes

The human olfactory system is intimately connected to the natural world. It involves a distinct avenue for perception via neuronal exposures to the airborne chemicals from nature, including those from vegetation, microbial communities, and bodies of water (*97*). Olfactory experiences of nature can include both pleasant and unpleasant odors and will be accompanied by different affective and autonomic responses accordingly. Natural olfactory environments—such as the smell of earth after rain or the aroma of pine trees during a forest walk—may instill a sense of connectedness and belonging within the larger natural world (*73*).

Reactions to natural odors contain dimensions of both affective and semantic valence—independent features that can be aligned or in conflict (*98*). Affective valence, the initial perception of (un)pleasantness, is relatively universal (*19*), possibly innate (*99*, *100*), and refers here to initial responses to nature based on perceptions of potential benefit or harm (e.g., sustenance versus toxins). Semantic valence, the later explicit judgment of (un)pleasantness, is partly influenced by lived experience, culture, and social factors—and includes higher-order, top-down information processing about the ways in which nature relates to well-being (e.g., an association of the smell of the sea with restoration due to a cultural practice). These judgments about the smells of nature may be shared or differ across populations and cultures (*44*, *45*, *101*).

### #3: Anthropogenic activity often negatively affects natural olfactory environments to the detriment of human well-being

Human action shapes natural olfactory environments in fundamental ways. Air pollution, climate change, deforestation, agricultural intensification, urbanization, and other dimensions of transforming and destroying nature continue to accelerate (*102*), negatively affecting natural olfactory environments (*103*–*105*). This includes influencing the production of levels of ozone and other impacts on sensescapes (i.e., the combined multisensory characteristics of an environment in a given geographical area at a specific point in time) (*106*, *107*) that are harmful to plants and animals, including human beings (*108*, *109*). Typically, the natural world exists in a state of photochemical balance with the atmosphere, with atmospheric oxidants continually cleaning the air of odorous signals and maintaining chemical spatial gradients (*95*). For example, although the amount of VOCs emitted by forests changes strongly in accordance with temperature, humidity, and light, interactions with other atmospheric chemicals keep ambient ozone levels in forested areas remarkably low and stable. However, emissions from anthropogenic sources (particularly NOx) can disrupt this balance and subvert the capacity of the biosphere to control the atmospheric environment (*110*). As these natural olfactory environments are degraded and destroyed through atmospheric and land-use change, fewer opportunities for experiencing a diverse range of odorants of nature are available. This change will likely have corresponding negative consequences for human well-being.

### #4: A better understanding of the relationship of human beings with natural olfactory environments can promote appreciation and revitalization of the natural world—and can thereby contribute to human well-being

As olfaction is typically undervalued and the benefits of natural settings are often overlooked (*111*), the loss of diversity of natural smellscapes is routinely ignored in large-scale assessments of the benefits of nature on human well-being. As urbanization continues, a shifting baseline and extinction of human experience with nature may be accompanied by a decreased capacity to recognize and appreciate the smells of the natural world (*112*, *113*). Studies have demonstrated how contact with more biodiverse natural settings is associated with greater human well-being (*114*, *115*), and this can extend to olfactory environments as well (*73*). With greater information about these benefits comes greater awareness of their loss, and the importance of actions to conserve and protect the olfactory environments of the natural world.

## CONCEPTUAL FRAMEWORK

**Fig. 1.**
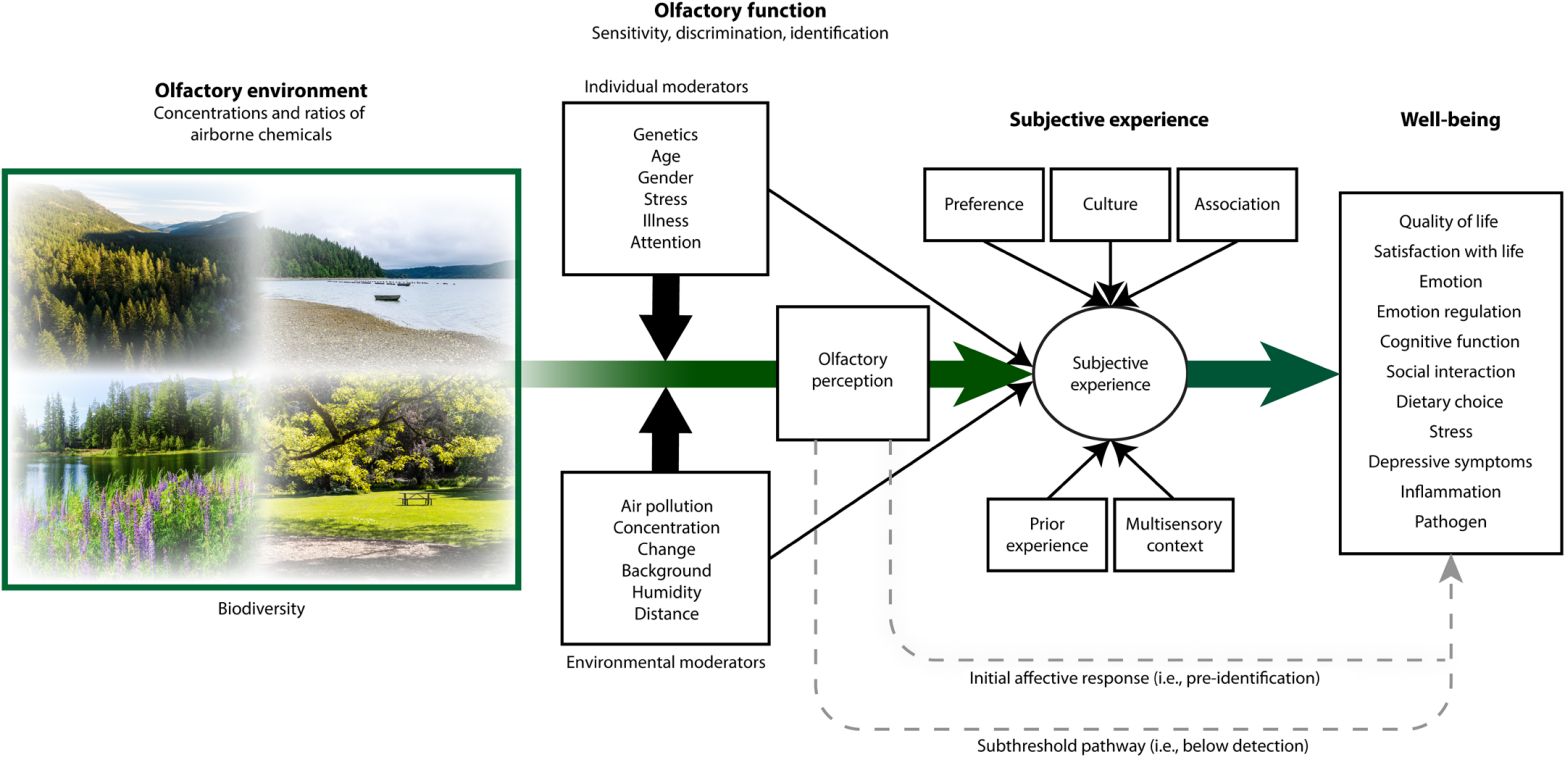
Conceptual framework of the pathway from exposure to natural olfactory environments to human well-being. The olfactory environment is characterized by the concentrations and ratios of airborne chemicals. Dimensions of olfactory function (i.e., sensitivity, discrimination, and identification) are influenced by a variety of individual and environmental factors, which together moderate olfactory perception. Subjective experience is a mediator through which olfactory perceptions lead to well-being outcomes. Relevant determinants of this experience include individual preference, culture, association, prior experience, and multisensory context. Other pathways to well-being include those that occur below the threshold of perception (i.e., subthreshold) and those that occur via initial affective responses that are suprathreshold but independent of top-down processes related to subjective experience. These components lead to a variety of well-being outcomes, from broader dimensions such as quality of and satisfaction with life, to emotional responses and emotion regulation, cognitive function, influences on behavior (social interactions and dietary choices), stress, depressive symptoms, (anti-)inflammatory processes, and effects from exposures to pathogens. Together, these outcomes are the result of subthreshold biochemical processes, initial affective responses, and subjective appraisals of odors from nature. A variety of other pathways mediate the relationship between olfactory environments and human well-being, although they are not illustrated here. Photos credit: University of Washington.

Building on these consensus statements, we propose a conceptual framework that recognizes the critical role that olfaction plays in the impact of the environment on human well-being (Fig. 1). While the focus of this framework is on the natural olfactory environment, the same principles and components can be applied to urban olfactory environments, including those that combine anthropogenic and natural emissions. We propose four components of this framework: (i) a characterization of the airborne chemical composition of the olfactory environment; (ii) determinants of sensitivity, discrimination, and identification that moderate perception of odors; (iii) determinants of the subjective experience that mediates the relationship of these olfactory perceptions with (iv) the well-being outcomes resulting from these processes.

It is important to note that not all factors involved in olfaction are illustrated here. Some are beyond the scope of this paper including, for example, the ways in which different levels of attention to odors can influence olfactory function itself (*116*), as well as a variety of other mediating pathways that lead from environmental exposures to well-being through the olfactory pathway.

### Characterizing the olfactory environment

Natural olfactory environments are generated from forests, meadows, deserts, wetlands, lakes, rivers, oceans, and many other settings and forms of life, from microbes to mammals to giant sequoias. The constituents of olfactory environments are determined in part by the types and abundances of biological species contained within the landscape. In addition, when volatile molecules from nature are released from their sources, they mix with the local atmosphere and undergo photochemical oxidation over the course of minutes to hours (*117*, *118*). These processes affect odor and scent concentration gradients, which have implications for the distances at which natural odorants are detectable. Individual airborne chemicals and the specific ratios and concentrations of mixtures in the air should be considered when characterizing the natural olfactory environment (see Box 3).

Box 3 **Additional measurement considerations.**

To characterize natural olfactory environments, interactions between natural ecosystems and the atmosphere should be considered (*273*). Forests emit an array of VOCs across a range of species as a function of light and temperature, with seasonal variation in strength and speciation (*274*). These compounds undergo oxidation in surrounding air through reactions with OH, O_3_, and NO_3 _(*275*–*278*). Mass spectrometry methods coupled to gas chromatography or proton transfer reaction mass spectrometry measures are typically used to measure terpene concentrations in the air (*279*, *280*), and the Model of Emissions of Gases and Aerosols from Nature has been used to estimate global terrestrial isoprene emissions (*118*).

Emissions from vegetation typically increase with temperature, often peak in the summer months, and can be affected by climate change (*281*–*284*). These considerations, along with oxidation, fluid turbulence, and rates of mixture with surrounding air, should be considered when modeling the presence of the VOCs from nature that exist in the air (*252*, *285*). In addition, many natural terpenes are chiral (*286*)—meaning that they exist in mirror image forms. Chirality expands chemical communication possibilities. The most abundant terpene measured in forests is alpha pinene. While (−) alpha pinene dominates the air of tropical forests, (+) alpha pinene is predominant in boreal forests (*286*). Although chirality may have an effect on insects (*287*), it is not yet known whether human beings react or respond to these changes.

Future efforts should also focus on expanding the tools available for measuring the presence of terpenes and other VOCs in ambient air to which human beings are exposed (e.g., through portable equipment that can be worn by individuals over the course of the day) and assessing absorbed dose of these VOCs in human beings (e.g., through serum collection and analysis) before, during, and after these exposures. In addition, accessibility to measurements of olfactory function should be increased. A lack of this availability often leaves individuals at a disadvantage to determine the level of their olfactory abilities. Without this insight, they may not know whether compensatory strategies to supplement lack of exposures or decreased processing of olfactory cues are necessary for their well-being.

### Moderators of olfactory perception of natural olfactory environments

Exposure to the odorants of nature may be deliberate or incidental. Whether the smells of a natural olfactory environment fall above or below thresholds of perception will be determined by a variety of individual-level factors related to olfactory function, such as genetics, age, gender, baseline levels of stress, illness, and attention (*119*). Environmental factors such as air pollution, concentrations of chemical mixtures and their rates of change, distinction from surrounding background, humidity, and distance to perceiver will also influence threshold levels, as will properties of the compounds themselves (*16*, *120*, *121*).

In addition, the specific mixtures of natural and anthropogenically generated airborne chemicals need to be considered. Through masking of odors from vegetation via oxidation and other processes, air pollution and elevated levels of ozone have been shown to disrupt chemical signaling that pollinators use to locate flowers (*105*, *122*–*126*). Relatedly, air pollution has been shown to disrupt olfactory function in human beings (*127*–*131*), as well as cause longer-term impacts through potential damage to cells in the olfactory epithelium (*132*, *133*)—all relevant factors to include when considering the olfactory perception of natural odorants. In addition, recent work has demonstrated that human beings may have evolved to be more sensitive to naturally occurring chemical species with lighter molecular weights or those that decay most rapidly, leading to differential odor detection thresholds (*134*, *135*). Mode of encounter (e.g., a burst versus a gradual appearance) is another relevant factor to include in this component (*136*).

### Subjective experience as a mediator

The subjective experience of olfactory environments mediates the pathway from perception to well-being and can be influenced by an individual’s preference, culture, association, prior experience, and the concurrent multisensory stimuli that provide additional context (*137*). Different depths of engagement with the natural world exist for different individuals—a factor that will likely influence this mediation as well (*138*). First person descriptions can provide insight into the role that smell plays in the subjective experience of natural landscapes (*73*, *139*). These experiences involve various levels of arousal, as well as affective and semantic valence, and can occur in the context of diverse activities—from prolonged park visits to smelling a garden or the sea from a window or rooftop (*57*). Human beings may also associate smell with natural landscapes differently, depending on specific types of nature. For example, sea and ocean are typically strongly associated with smell, but streams and rivers are often not (*140*). Some aspects of the moderators of olfactory perception may also influence subjective experience (e.g., genetics, age, and gender).

### Well-being outcomes

Together, although the evidence base is still growing, exposure to nature via the olfactory pathway may affect a variety of different types of well-being. Impacts associated with olfactory processing of natural airborne chemicals can lead to beneficial affective outcomes or, in some cases, adverse ones (e.g., unpleasant memories associated with a negative prior nature experience). The association of olfaction with quality of and satisfaction with life (*29*–*33*) may extend to experiences of the natural olfactory environment that are tied to connection with the natural world, individual and community identity, and sense of place (*73*, *76*, *77*). Studies have found that nature contact is associated with emotion, emotion regulation, and cognitive function (*141*, *142*), and the substantial tie between olfaction and these outcomes as well (*28*, *143*, *144*) supports the possibility that the olfactory pathway may play an important role in these documented impacts from nature contact (*145*).

Research has shown that odors influence social interactions [e.g., tendencies to cooperate, select friends and mates (*40*, *146*), shake hands (*147*), or influence parent-infant bonding (*148*, *149*)]. It is therefore possible that natural olfactory environments may contain molecules that influence these interactions, including via subthreshold pathways that influence social preferences or aggression (*150*, *151*). Orthonasal smelling yields anticipation for macronutrients, modifies food selection, and regulates appetite, even in the absence of physiological hunger. As with other odors that influence dimensions of dietary choices [e.g., food preference and quality of diet (*152*, *153*), appetite (*28*), food flavor and enjoyment (*154*, *155*)], those from nature may have a substantial influence on these choices by causing certain foods to be more appealing. For example, the scents of edible plants in a garden may increase the desire for fresh vegetables and fruits over processed options.

As with other affective outcomes, many studies have demonstrated an association of nature contact with stress responses and depressive symptoms (*57*). Evidence from olfactory function research (*156*–*158*) supports the notion that the olfactory pathway may play one explanatory role for these effects and associations. Last, research from Shinrin-yoku supports the association of nature contact with anti-inflammatory outcomes via the olfactory pathway (*80*, *84*).

Future studies will help to inform this emerging body of evidence. With these investigations, it will be important to consider additional moderators related to conscious appraisals of smells of nature, as well as subthreshold biochemical pathways to well-being from natural olfactory environments. Relatedly, there is growing evidence that the constituents of air pollution negatively affect health through inhalation, including via the olfactory pathway (*159*–*162*) (see Box 4). Some of these constituents, impacts, and pathways may be relevant to future work that investigates the effect of natural olfactory environments on human well-being.

Box 4. **Effects of air pollution on well-being.**

Evidence from a variety of disciplines provides insight into the mechanisms through which the air that human beings breathe affects well-being. These include exposures to particulate matter (e.g., fine and ultrafine) and other harmful constituents of air pollution that come from tailpipe emissions, fuel refineries, transportation corridors, wildfire smoke, and other sources—all of which can affect well-being by increasing the risk for cardiovascular disease, dementia, anxiety, and depression via oxidative stress and other inflammatory mechanisms (*160*, *288*–*290*). It is also now recognized that air pollution exposure is a risk factor for diseases such as Alzheimer’s disease (*291*). Other support for the adverse impacts of human-generated airborne toxins comes from the fact that individuals living near major highways are at higher risk for Alzheimer’s disease, Parkinson’s disease, and multiple sclerosis (*292*).

Beyond the direct effects on human well-being linked to neurochemical and neuroendocrine changes, exposure to air pollution can also lead to other behavioral impacts. Specifically, increased inflammatory cytokine activity and systemic inflammation can be associated with “sickness behavior,” via a CNS pathway, characterized by withdrawal, symptoms related to depression, and hypervigilance or an increased awareness of threats (*293*–*295*).

## PRACTICAL APPLICATION

With continual integration of future findings, this conceptual framework can begin to inform decision-making efforts that account for the repercussions of landscape change for human well-being via associated impacts on natural olfactory environments. This approach follows a preliminary version of past examples in ecosystem service modeling and scenario generation, in which different potential futures are modeled and compared to inform decisions (*57*, *163*–*167*). Today, this approach underpins the transformation to nature-positive, inclusive development pathways being pioneered by cities, countries, multilateral development banks, and other partners (*164*, *168*).

Recent efforts and evolving methodologies in mapping smells through the use of field olfactometry (*169*) may contribute to the spatial specificity of these scenarios as they relate to olfactory environments, as well as diffusion functions that account for distance decay from plants to individuals and other factors related to the “geography of smell” (*58*, *69*, *170*, *171*). Analogous efforts exist with respect to examining soundscapes, including natural ones. For example, models that describe natural soundscape quality have been developed using specific indicator metrics that allow for quantification, mapping, and visualization of soundscapes—information that can then be integrated into development decisions (*172*). Eventually, it will be possible to create an array of projections (i.e., three-dimensional maps of smellscapes) that include both the adverse effects of air pollution and the beneficial effects of natural odorants when considering the repercussions of alternative development scenarios on human well-being (*66*, *173*).

Together, these components allow for the incorporation of spatial and temporal factors in modeling the impacts of landscape change on olfactory environments. Future work to refine this model should continue to integrate interdisciplinary research from atmospheric chemistry, epidemiology, exposure science, neurobiology, ethnography, Indigenous Knowledge, and environmental psychology. To assure ecological validity and best reflect human-nature interactions, these potential avenues of research should examine the mechanisms of the olfactory pathway in the multisensory contexts in which they exist in the natural world.

## FUTURE DIRECTIONS

Many understudied areas exist for future research on the impact of nature on human well-being through the olfactory pathway. Below, we outline some of these potential intersections and research frontiers.

### Nature, enriched environments, and olfactory function

Loss of sense of smell (i.e., anosmia) (*174*) has been associated with decreased well-being through risk of exposure to chemical hazards and the inability to fully experience a variety of stimuli, social connections, and environments (*28*, *175*, *176*). Reduced olfactory function can also serve as an early indicator of such neurological diseases as Alzheimer’s disease and Parkinson’s disease and can be associated with depression (*30*, *32*, *175*, *177*–*183*). Idiopathic olfactory loss may precede the symptoms of neurodegenerative diseases by several years and has a distinctive clinical pattern in comparison to other instances of dysosmia (i.e., smell hallucinations or phantosmias). Relatedly, studies have demonstrated that improvements in smell after prior loss are associated with higher levels of well-being and reduced depressive symptoms (*184*–*187*).

In recent years, a substantial portion of the global population has experienced an impaired sense of smell on at least a temporary basis due to COVID-19 infection—and the subsequent consequences of this on well-being are now being investigated (*188*–*192*). In addition to disease, there is evidence that odor deprivation and consistent exposure to sterile, odor-deprived environments may diminish human olfactory function (*193*–*195*). For example, clean room workers who spent significant portions of each day in rooms deliberately deprived of odorants exhibited decreased ability to discriminate between odors, as well as an elevated odor perception threshold. These effects were exacerbated with longer durations of time in the clean room environment (*196*). Conversely, enriched olfactory environments have been shown to provide support for a range of neurological challenges, including behavioral and cognitive outcomes evident in autism spectrum disorders (*197*). Findings like these suggest that exposure to a wide range of natural smells could act as a training mechanism—continuously maintaining and improving the olfactory system’s functional capabilities and, through this effect on function, contribute to increased human well-being. This is a vital arena for future research.

### Environmental psychology theory

Studies that are motivated by two dominant theories from environmental psychology—attention restoration theory (ART) (*59*) and stress reduction theory (SRT) (*198*)—have focused primarily on the visual pathway as the one through which natural stimuli have a restorative effect on cognition and affect. However, the principles underlying these theories can be applied to natural olfactory stimuli as well (*73*). This approach may offer potential insight regarding causal mechanisms underlying the restorative effects of natural odors on human well-being.

For example, ART posits that individuals have a limited capacity for directed attention, a resource that allows for focus and concentration on a specific set of stimuli, while blocking out competing distractions (which are often present to a greater degree in urban environments). Natural environments are hypothesized to restore directed attention insofar as they contain qualities that are “softly fascinating” and give a sense of “being away,” among other factors. In these types of restorative environments, involuntary attention is engaged, which allows for a replenishment of directed attention and subsequent improvements in cognitive function (e.g., short-term working memory, concentration, and impulse inhibition) (*199*–*201*). These findings align with studies that reveal an association of greater olfactory function and training with increased cognitive function (*184*, *185*), as well as the literature that demonstrates the relationship of decreased olfactory function with decreased cognitive function and increased cognitive impairment (*29*, *202*–*205*).

With respect to smellscapes, it may be the case that many urban environments present a multitude of odors—a large proportion of them anthropogenically generated—that tax our directed attention as we attend to odors most relevant to specific situations and demands. As a human being moves through an urban landscape, there may be frequent and intense demands upon olfactory attention (e.g., vehicle exhaust, pizza, trash, cigarette smoke, and sewage). This contrasts with many natural environments, in which smellscapes are more constant and only sharp changes on the otherwise slowly changing chemical background are perceived. Movement through a natural landscape may therefore be less demanding on our olfactory attention. Important research foci include causes of odor fatigue (*206*), mental fatigue from odor exposures (*207*), and specificities of the environment that affect olfactory awareness and attention (*64*, *137*) to examine whether human beings experience odor fatigue in urban environments. These studies could then investigate whether olfactory attention restoration can be fulfilled directly by exposure to natural olfactory environments in ways that are similar or complementary to the restoration that occurs via the visual pathway in ART studies.

Studies have demonstrated the potential of nature exposure to reduce stress in compelling ways (*208*). Roger Ulrich’s psychoevolutionary SRT posits that many natural environments restore and reduce acute and chronic stress in human beings through an initial, precognitive affective response and subsequent engagement of the parasympathetic nervous system. This theory may be related to initial affective responses to the smells of nature—those that have an impact on our well-being through a prelinguistic pathway, independent of our later semantic processing of these effects. Future research could examine this further by assessing the degree to which odorants from nature result in affective benefits through initial responses, and how these reactions do or do not differ from later subjective experiences (and resulting impacts on affect).

### Biodiversity

The diversity and abundance of life strongly influence how and how well ecosystems support human well-being (*209*, *210*). Biodiversity is also fundamental to the diversity of the human lived experience, including olfactory perceptions (*211*, *212*). Contact with more biodiverse nature has been shown to benefit human well-being to larger degrees than less biodiverse nature (*115*, *213*), and olfaction is an understudied potential pathway through which these benefits may occur (*73*). In addition, the plasticity of the olfactory system over the lifetime of an individual – and through the evolution of species over time – implies that as environments change, olfactory function may change as well.

Emerging evidence suggesting that anthropogenic ecosystem change may influence neurobiology (*214*) merits further attention to investigate whether decreased biodiversity of olfactory environments harms cognitive function and neurodevelopment in human beings. A related frontier concerns whether a narrowing or broadening of available odors of nature is associated with a contraction or expansion of corresponding vocabularies of smell, interactions with nature, and outcomes for human well-being (*112*, *215*, *216*). With respect to decision-making in this context, recent work demonstrated the importance of framing biodiversity in terms of positive or negative change (versus absolute numbers) and showed that positive emotional responses to gains in biodiversity may be greater in magnitude than negative emotional responses to biodiversity losses (*217*). These and related future findings can inform interventions designed to motivate protection and restoration of natural olfactory environments.

### Breath

Great value would come from investigating the role of other physiological mechanisms that underlie the impacts of nature contact on human well-being via the olfactory pathway. For example, smells of nature may affect respiration in beneficial ways. In some languages, the concept of smell is closely linked with the concept of breath (*218*) and accounts from the Anangu Aboriginal people of Western Australia describe the post-rain smell of the desert as one that encourages ease of breathing (*219*). Olfactory perceptions of safety in the natural environment may signal to the central nervous system (CNS) that it is safe to breathe more deeply—a behavior that has been shown to reduce self-reported stress and levels of cortisol and slow heart rate (*220*). Imagery of pleasant versus unpleasant odors has been shown to cause deeper inhalation through the nose (i.e., “sniffs”) (*221*), and future research should test these effects using natural stimuli specifically.

### Nasal microbiome

Last, the potential role of the nasal microbiome in mediating the relationship between nature exposure and human well-being merits further investigation (*222*). This community, consisting of billions of microbes, exists under persistent interaction with other microbial life as well as various mixtures of biogenic and anthropogenic airborne chemicals from the environment (*223*). Differences in nasal bacterial community composition and lower nasal microbiome diversity have been shown to be associated with decreased ability to discriminate odors (*224*, *225*). These results suggest that the nasal microbiome could play a part in shaping an individual’s sense of smell. There is also emerging evidence that nasal microbiome composition may vary between those who grow up in a rural environment versus an urban one (*226*). Future research should build upon this foundation to explore the ways in which the composition and function of this microbiome may be influenced by natural olfactory environments.

## CONCLUSION

Human beings are chemosensory communicators (*40*), a capacity we share with many other species in the natural world (*227*). We live in a reciprocal relationship with nature through interactions in the airborne chemical environment—a context that our actions can degrade (e.g., generating air pollution and losing forest/grassland cover via climate change–induced wildfire) or enhance (e.g., creating natural urban greenspaces and conserving large natural areas). Given rapid environmental change, it is urgent that we increase attention to the critical contributions that natural environments make to human olfactory experiences and well-being.

We also underscore the importance of including a full range of cultural contexts in this emerging field. As humanity becomes ever more urban and experiences ever less nature (*57*), we are cut off from an evolutionary library of olfactory experience. We are only beginning to learn about the complexities of the functioning of the human olfactory system. This includes the fact that olfactory receptors exist in other parts of the body outside of the nose, such as the skin, liver, prostate, and muscles (*228*). It unknown whether their expression levels are correlated with those within the olfactory neuroepithelium and the degree to which they also influence human health and well-being.

Understanding more about natural olfactory environments is important not only because of associations with human well-being but also because protecting chemosensory communication is core to protecting nature. Outside of an anthropocentric focus, there is a critical importance of the “volatilome” to the functioning of nature itself, and these concerns should inform preservation and conservation efforts (*229*). Recent activity to protect soundscapes and reduce light pollution in national parks could be expanded to include smellscapes, as has recently been done in certain parts of France through sensory law (https://www.assemblee-nationale.fr/dyn/15/dossiers/definition_protection_patrimoine_sensoriel_campagnes).

Together, the material reviewed in this paper provides a foundation for developing and implementing activities that account for the role of the olfactory pathway. We know very little about the intricacies and interactions that occur within natural olfactory environments. Their protection should be prioritized as we continue to uncover the complex ways in which they support the flourishing of the human and more-than-human world.
